# Locally Controlled Sensing Properties of Stretchable Pressure Sensors Enabled by Micro-Patterned Piezoresistive Device Architecture

**DOI:** 10.3390/s20226588

**Published:** 2020-11-18

**Authors:** Jun Ho Lee, Jae Sang Heo, Keon Woo Lee, Jae Cheol Shin, Jeong-Wan Jo, Yong-Hoon Kim, Sung Kyu Park

**Affiliations:** 1 Department of Electrical and Electronics Engineering, Chung-Ang University, Seoul 06974, Korea; junofet@gmail.com (J.H.L.); lkw9941@gmail.com (K.W.L.); tlswo0627@naver.com (J.C.S.); 2 School of Advanced Materials Science and Engineering, Sungkyunkwan University, Suwon 16419, Korea; heojs38@gmail.com; 3 Electrical Engineering Division, Department of Engineering, University of Cambridge, 9 JJ Thomson Avenue, Cambridge CB3 0FA, UK; jzw0108@gmail.com; 4 SKKU Advanced Institute of Nanotechnology (SAINT), Sungkyunkwan University, Suwon 16419, Korea

**Keywords:** pressure sensor, stretchable sensor, wearable electronics, artificial skins

## Abstract

For wearable health monitoring systems and soft robotics, stretchable/flexible pressure sensors have continuously drawn attention owing to a wide range of potential applications such as the detection of human physiological and activity signals, and electronic skin (e-skin). Here, we demonstrated a highly stretchable pressure sensor using silver nanowires (AgNWs) and photo-patternable polyurethane acrylate (PUA). In particular, the characteristics of the pressure sensors could be moderately controlled through a micro-patterned hole structure in the PUA spacer and size-designs of the patterned hole area. With the structural-tuning strategies, adequate control of the site-specific sensitivity in the range of 47~83 kPa^−1^ and in the sensing range from 0.1 to 20 kPa was achieved. Moreover, stacked AgNW/PUA/AgNW (APA) structural designed pressure sensors with mixed hole sizes of 10/200 µm and spacer thickness of 800 µm exhibited high sensitivity (~171.5 kPa^−1^) in the pressure sensing range of 0~20 kPa, fast response (100~110 ms), and high stretchability (40%). From the results, we envision that the effective structural-tuning strategy capable of controlling the sensing properties of the APA pressure sensor would be employed in a large-area stretchable pressure sensor system, which needs site-specific sensing properties, providing monolithic implementation by simply arranging appropriate micro-patterned hole architectures.

## 1. Introduction

Recently, mechanically resilient pressure sensors have been gathering considerable interest in artificial skins [1,2,3,4,5], health monitoring systems [6,7,8,9,10], and wearable electronics [11,12] owing to their versatile detectability such as detection of bio-signals and body movements. The pressure sensors can be categorized into capacitive [13,14,15,16], piezoelectric [17], triboelectric [18,19,20,21,22], and piezoresistive [23,24,25,26,27,28] type sensors, according to their specific operating principles. Although other pressure sensors could be self-powered and exhibit high performance, those sensors typically indicate low sensing detection range and low sensitivity, resulting in inaccurate determination of the actual mechanical stimuli [29,30,31]. Therefore, among various types of sensors, the piezoresistive sensors that are operated by changing the resistance of sensing materials have been extensively explored because of the simple data acquisition and fabrication process and low power consumption. Accordingly, they have more potential to be integrated with various other types of mechanical stimuli sensory systems than other types of pressure sensors. Especially, in order to enhance their performances, two representative strategies were mainly used. The first approach is to adopt porous structures such as a hollow sphere and sponge shapes as sensing materials for the pressure sensors [32,33,34]. The second approach is to utilize nano-/micro-structures such as pyramid structures and micro-cracks, providing enhancement of the response against external mechanical stimuli [35,36,37]. However, although the pressure sensors using these effective methods exhibited high sensitivity and fast mechanical responses to various physiological-/activity-signals, it still remains a challenge to develop a stretchable pressure sensor that can simultaneously allow high stretchability, good linearity, and a wide sensing range in a skin-adaptable large-area structure. To date, for demonstrating a highly stretchable/flexible pressure, a lot of studies related to the enhancement of deformability have been performed using various methods such the use of polyimide (P.I) films, a liquid metal embedded in polydimethylsiloxane (PDMS) matrix, and conductive nanomaterials [27,38,39,40,41]. However, in terms of practical wearable applications for skin-attachable health monitoring systems and soft robotics, these approaches are not enough to obtain appropriate stretchability (up to 40%), and are bulky devices with a thickness of above 2.5 mm. To address the issue above, Z. Wang et al. introduced a 3D printing technology, resulting in high stretchability (up to 50%) and a wide sensing range (10~800 kPa) [42]. Despite the significant improvement in device performance, the sensor was unable to detect pressure in the stretched state, which may induce inaccurate data on pressure stimuli. Thus, an efficient and competitive approach is definitely required to achieve high deformability, and precisely detecting output signals originating from pressure stimuli.

Here, we demonstrate a highly stretchable piezoresistive type pressure sensor using silver nanowires (AgNWs) as stretchable electrodes and a micro-patterned polyurethane acrylate (PUA) film as both a spacer and a sensing control layer simultaneously. Particularly, utilizing a micro-patterned hole structure in the PUA spacer and appropriately designing the patterned hole area, the sensing characteristics of the pressure sensors such as the sensitivity, pressure sensing range, and linearity could be effectively controlled. A stacked AgNW/PUA/AgNW (APA) structural design for highly stretchable pressure sensors was able to allow control of the sensitivity in the range of 47~83 kPa^−1^ as well as the sensing range from 0.1 to 20 kPa. In addition, to ensure the viability of the developed pressure sensor, various stretching tests were conducted in a variety of conditions, verifying the act of the devices operable up to 40% of stretching without significant degradation. Consequently, the results reported here imply that sensitivity, sensing ranges, and linearity of the locally controlled pressure sensors could be controlled by simply adjusting the hole size (highly sensitive devices can be located at the area where high sensitivity is required, and relatively low sensitive and large sensing range devices can be formed at the other area) for boosting the device performance corresponding to environmental conditions. Additionally, the APA pressure sensor can be stretchable with uniform pressure sensitivity, which enables them to be suitable for various wearable electronic applications. These features enable efficient systems to be implemented in human skin or robot systems by organizing cells with different sensitivity in a single sensory system. Moreover, the APA sensors would offer a general route to a position-dependent large-area pressure sensor system with marginal system complexity via CMOS compatible conventional photolithography and bottom-up processing.

## 2. Materials and Methods

### 2.1. Preparation of Stretchable Hole-Patterned Pressure Sensor Device

For the fabrication of the substrate, PDMS (Sylgard 184, Dow Corning), the ratio of base to curing agent was 10:1 in weight, spin-coated on the 5 × 5 cm glass at 500 rpm in 30 s, and cured at 75 °C in 2 h. Followed by O2 plasma treatment, for the bottom stretchable electrode, AgNWs (average length of 15 μm, an average diameter of 30 nm, 1% isopropanol dispersion, Ditto Technology Co.) were spin-coated on the PDMS substrate using consecutive two-step coating processes with different spin-speeds (500 rpm and 1000 rpm) to achieve more uniform AgNW films on the hydrophobic surface of the PDMS. Then, for the selective electrode line, the AgNW film was patterned by photolithography (UV with 365 nm) with the metal etchant selectively masked by positive photoresist at 30 s. After development, on the bottom electrode layer, the urethane acrylate solution (UA) was spin-coated at 200 rpm for 50 s and then baked at 70 °C for 20 min. Then, the UA-coated film was exposed to ultraviolet (UV) light (with 365 nm) to form the stretchable polyurethane acrylate (PUA) [43]. Moreover, using a photomask with various hole sizes (D-10, 50, and 200) and shapes (single and multi-mixed), the cross-linked PUA was patterned to desirable shapes and unexposed UA was easily removed by an acetone-rinsing process. As a result, the PUA film with patterned holes was fabricated on the bottom electrode, forming an insulation layer for the AgNWs-based pressure sensor. Subsequently, the AgNWs top electrode was spin-coated and patterned with the same method as the bottom electrode layer. Finally, the PDMS as the protection top layer was molded with the same method with substrate layer.

### 2.2. Analyses of Pressure Sensing Characteristics

The electrical conductivity of AgNW bottom electrode was measured using a resistance-meter and four-point probe measurement system. The relative change in current was measured using a semiconductor parameter analyzer (Agilent 4156C, Agilent Technologies). The response and relaxation time to pressure was determined as the minimum time from initial state to pressure stimuli using the cyclic loading measurement data. For the dynamic measurement of the sensor, a measuring system comprised of a dual channel source meter (Keithley 2636B) connected to a data acquisition system (DAQ; SnM). The customized gauge force for the precise measuring pressure and response test was used. Using this measurement system, the acquisition of pressure sensing data is possible by sequentially reading the resistance from each sensor (Figure S11).

## 3. Results and Discussions

### 3.1. Fabrication and Structure of APA Pressure Sensor

**Figure 1:**
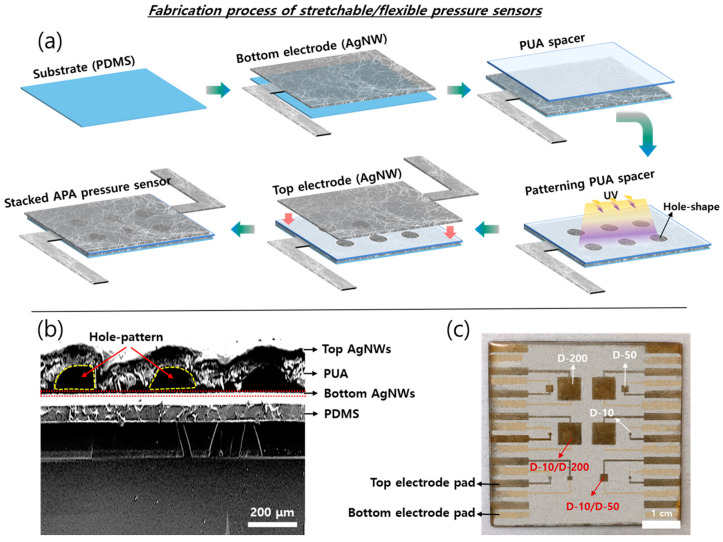
The schematic of the fabrication process and structure of the stretchable sensor. (**a**) A schematic of fabrication process of controllable and stretchable pressure sensor. (**b**) The scanning electron microscopy (SEM) image of the cross-section view of the patterned hole sensor layer. The sensor comprises two stretchable silver nanowire (AgNW)-layers as top and bottom electrodes, polyurethaneacrylate (PUA)-layers as a sensing pattern and insulation layer, and polydimethysiloxane (PDMS) as a bottom substrate and moulding. (**c**) A photograph of the stacked AgNW/PUA/AgNW (APA) piezoresistive type stretchable pressure sensor.

The fabrication procedure of a stacked AgNW/PUA/AgNW (APA) piezoresistive type stretchable pressure sensor is illustrated in Figure 1a. Firstly, a highly stretchable bottom AgNW film was formed on an intrinsically stretchable PDMS substrate using a spin-coating process and then patterned by conventional photolithography processes, forming a desired structure/size (extended electrodes line for measurement in Figure 1b). Afterward, the photo-patternable UA serving as a spacer or buffer layer was spun over the AgNW bottom electrode and, subsequently, the UA layer was selectively patterned and cross-linked to PUA by exposing ultraviolet (UV, 365 nm) for 8 s with a photomask, which enabled the formation of microstructure with various sizes and shapes. In this study, in order to investigate the effect of the pressure sensor’s performances on different hole size and thickness of the PUA layer, we used circular hole diameters (D) of 10, 50, and 200 µm and the thickness (T) of 300, 500, and 800 µm. For the pressure sensor unit with a stacked APA structure, the AgNW film for a top electrode was formed on the desirably patterned PUA layer and then patterned using the same method with the bottom electrode. Finally, the whole devices were molded with additional PDMS layer to achieve high reliability during stretching and severe mechanical operation. The total thickness and the size of the stacked APA pressure sensor were less than 1 mm and 50 × 50 mm^2^, respectively, which can be suitable for applying to wearable electronics. More detailed fabrication procedure and materials are described in the experiment section. Figure 1b show the fabricated APA pressure sensor and a cross-sectional scanning electron microscopy (SEM) image of a micro-patterned pressure sensor including 200 µm diameter hole structures, respectively, showing that the APA pressure sensor has several distinct layers in the stacked sandwich structure including a PDMS substrate (~10 µm), very thin AgNW electrodes (~100 nm), and a hole patterned PUA layer (~100 µm) as designed. Moreover, the picture of the fabricated APA pressure sensor with various single/multi-mixed holes with different sizes is shown in Figure 1c.

### 3.2. Working Mechanism and Analysis of Static Electrical Properties with Different Diameter of Hole Pattern

**Figure 2:**
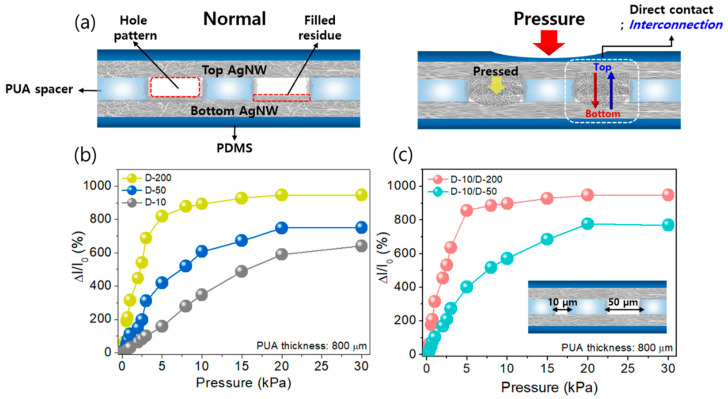
The sensing mechanism and analysis of static response to pressure and the electrical characteristics. (**a**) Simple schematics of the pressure sensing mechanism. (**b**,**c**) The relative current change of single hole patterned pressure sensor (D-10, 50 and 200 µm) and multi-mixed holes patterned pressure sensor (D-10/50 and 10/200) in a large pressure range (0.1–30 kPa).

In general, the sensing performances of pressure sensors are highly dependent on a specific structural configuration of sensor devices. Herein, we focused on both a structural design of the PUA spacer that separates two (top and bottom) electrodes and a sensing area that can control the conductive pathway of AgNWs. Particularly, the size of the patterned hole and the thickness of the PUA film are key factors to achieve good piezoresistive characteristics of the pressure sensors. Figure 2a shows the working mechanism of the stacked APA sensor under pressure. Actually, note that a small amount of the AgNWs would fill the hole, and then a very thin AgNW film would be formed inside the hole, resulting in a thickness increase of the bottom AgNW electrode at hole-pattern regions, as shown in Figure 2a [44]. Moreover, as shown in Figure 1b, it was observed that the hole-patterns are still maintained after the formation of the AgNW top electrode. As no pressure is applied, direct contact between the top and bottom AgNW electrodes is effectively prevented by the PUA spacer, resulting in high contact resistance. However, there are some imperceptibly fine conducting pathways that exist between the electrodes because the AgNWs were coated along the walls of the PUA hole patterns during the coating process of the top AgNW electrode, as shown in Figure S1. On the other hand, when sufficient external pressure is applied to the sensor, the upper AgNW electrode is pressed and the direct contact arising from the interconnection between the two AgNW electrodes occurs through the hole pattern, which causes a decrease of contact resistance. This result is attributed to higher Young’s modulus (E) of the PUA compared with the PDMS (EPUA = ~320 MPa, EPDMS = ~3 MPa). Actually, the pressure applied to the sensor could be easily delivered to the AgNW electrode coated on the PDMS, while the PUA layer maintains the shape of the hole pattern and the gap distance between the electrodes. As a result, the contact resistance of the stacked APA pressure sensor is repeatedly varied by a generation/degeneration of the interconnection between the two AgNW electrodes under pressure. However, it will be saturated when a certain level of pressure is reached, causing the pressure-independent characteristics.

To analyze pressure sensing characteristics of the stacked APA sensor with micro-patterned holes, current variation (ΔI/I_0_) of the sensors having different hole sizes (10, 50, and 200 µm) and PUA’s thickness of 800 µm was measured under different pressures (0~0 kPa), as shown in Figure 2b. As a result, all samples showed that the relative change in current gradually increased with increasing pressure, regardless of the hole diameter sizes, while a large current variation and high sensitivity were found with increasing the sizes. In the case of small hole dimeter size (10 µm, D-10), the sensor exhibited the sensitivity of 29.4 kPa^−1^ and good linearity up to 20 kPa. However, even though the sensors with D-50 and D-200 showed improved sensitivity of 60.7 kPa^−1^ (0~10 kPa) and 163.4 kPa^−1^ (0~5 kPa), respectively, the detection linearity was significantly degraded, which indicates that the sensing performances of the sensor can be controlled by tuning the hole diameter size. In general, a polymer-based pressure sensor has a material limitation that can cause the change of linearity to the different pressure range, so the APA sensor also shows the different sensitivity depending on the hole size and the thickness of spacer. Consequently, there are some uncertain areas where it is difficult to set an accurate standard for sensitivity. Therefore, the sensitivity of each range and the linearity were calculated and presented using the program tool. Note that detailed information on linear fitting is shown in Figure S2. The properties (sensitivity, sensing range, thickness, and hole density) of the APA pressure sensors are summarized in Table S1. Moreover, in addition to the hole diameter size, the effect of multi-mixed holes with different sizes (D-10/D-50 and D-10/D-200) was investigated. Figure 2c shows the corresponding pressure sensing characteristics of the sensors with the multi-mixed holes. From the results, it was found that the sensitivity tends to be dependent on the properties of the sensor with large hole patterns owing to the fact that the AgNW interconnections serving as a source for the change in contact resistance of the sensor are sensitive to larger-sized holes than small ones. Moreover, compared with the sensors with single-sized holes, the linearity in the low-pressure range and the sensitivity at the same pressure range is improved by using the multi-sized ones (Table S1). Particularly, the sensors with the D-10/D-50 and D-10/D-200 holes exhibited the improved sensitivity of 38.86 kPa^−1^ in the pressure range of 0~20 kPa and 171.5 kPa^−1^ in the pressure range of 0~5 kPa, respectively. The details with linear fit are shown in Figure S3. Generally, in the large size of hole pattern sensor, because the direct contacts between top and bottom electrodes come faster (in ~10 kPa), the sensitivity is saturated in a relatively small pressure range compared with the other small size of the hole pattern sensor. In conclusion, the linear sensitivity (calculated in Figures S2–S4) tends to increase following the hole size in the PUA. The tendency of linear sensitivity in different hole size and patterns is indicated in Figure S5.

### 3.3. Analysis of Static Electrical Properties with Different Thickness of PUA and Dynamic Sensor Performance

**Figure 3:**
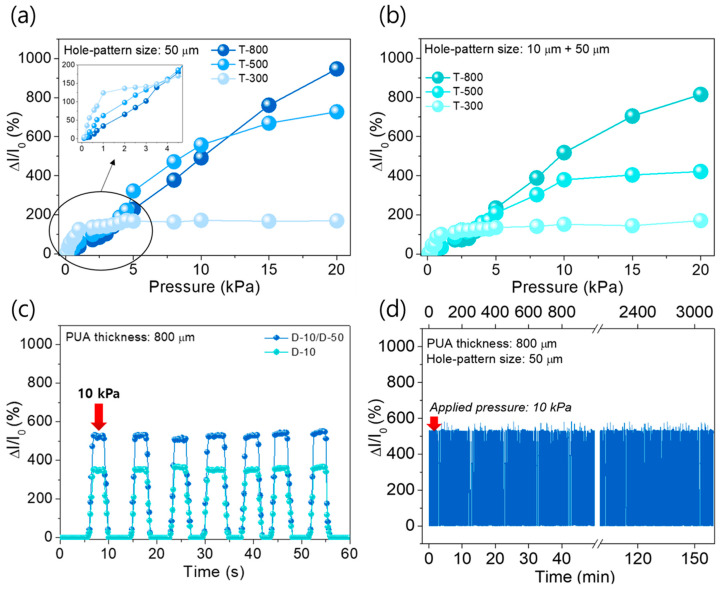
Analysis of static response and dynamic response to pressure with different PUA thickness, stretched state. (**a**) The relative current change of D-50 pressure sensors with different PUA thickness (300, 500, and 800 μm) up to 20 kPa. The inset shows the small pressure range (up to 4.5 kPa). (**b**) The relative current change of D-10 + 50 pressure sensors with different PUA thickness (300, 500 and 800 μm) up to 20 kPa. (**c**) The dynamic responses of D-50 and D-10 + 50 pressure sensor in 60 s. The sensors pressed on 10 kPa in 5 s and released repeatedly through pressing weight. (**d**) The cyclic test data of the pressure sensor for steady pressure sensing stability at a frequency of 0.1 Hz (about 3200 cycles).

Consequently, the pressure sensing characteristics such as the sensitivity and the sensing range were largely dependent on the hole size and their composition with different sizes. However, the properties of the stacked APA sensors might be unsatisfactory for certain applications because of poor linearity and/or narrow sensing range, except for the sensor with the D-10. For instance, in robotic applications, pressure sensors should have high sensitivity and wide sensing range as well as good durability against the external stimuli. Accordingly, the enhancement of the sensor’s performances is generally needed to broaden a restricted range for practical applications. In this regard, in order to improve the performance of the pressure sensor, we focused on the PUA thickness as another design factor that can affect the pressure sensing properties. In fact, with a thick PUA spacer, higher pressure is required to make direct contact between the top and bottom AgNW electrodes. To evaluate the influence of PUA thickness on the sensing behavior, we fabricated the stacked APA pressure sensors with different PUA thickness (T) of 300, 500, and 800 µm and the fixed hole size (D-50); then, the corresponding relative change in current as a function of pressure was analyzed. As shown in Figure 3a, the sensing linearity was improved as the PUA thickness was increased. The sensor with the PUA thickness of T-300 had a very narrow sensing range up to about 2.5 kPa, while the extended sensing ranges of ~10 kPa and 20 kPa were exhibited for the sensors with T-500 and T-800, respectively. Note that detailed information on linear fitting is shown in Figure S4. In addition, the sensitivity was deteriorated with the increasing PUA thickness from 300 μm to 800 μm (Table S1). This is because the increased PUA thickness requires high pressure to reach the percolation threshold in the stacked APA structure. In other words, although a thin PUA spacer induces high sensitivity, it is difficult to expand their sensing range because of a low percolation threshold value. Additionally, we have investigated the effect of multi-sized holes (D-10/D-50) on the sensor with different thicknesses of the PUA space, as shown in Figure 3b. Based on the results, it was observed that the relative current change (ΔI/I_0_) is considerably decreased compared with those with a single D-50 hole pattern, which is attributed to the addition of a D-10 hole pattern that could cause a low response to pressure (Figure 2c). Consequently, the pressure sensing range tends to increase following the thickness of the PUA. The tendency of the pressure sensing range in different thicknesses of PUA is exhibited in Figure S6. In addition, the response time and the operational stability of the stacked APA pressure sensors with the single hole D-50 and the multi-mixed hole pattern consisting of D-10, D-50, and T-800 are evaluated. The dynamic current response to applying the pressure of 10 kPa was measured at the frequencies of 0.1 Hz and 1.0 Hz, as shown in Figure 3c and Figure S7, respectively, exhibiting a stable pressure sensing performance with the response and recovery time of ~100 ms and ~110 ms, respectively. It indicates that this result may allow real-time monitoring against pressure in applications such as wearable devices and electronic skins. In addition, the APA pressure sensors showed a good reversibility response of ΔI/I0 to the applied step-up and step-down pressures from 0 to 20 kPa, with a 2 s intervals in each step, as shown in Figure S8. In general, for wearable pressure sensors, transparency and flexibility are important factors. Accordingly, we additionally performed the transparency of the APA sensor, as shown in Figure S9. As a result, the transparency of ~60% was exhibited at the visible range (380–780 nm), which is suitable for wearable electronics and robot application. Moreover, for the sensor with D-50 and T-800, the cyclic pressure test was conducted with an applied pressure of 10 kPa to verify the durability of the pressure sensor for more than 3200 cycles, as shown in Figure 3d. The result reveals that the relative change in the current of the pressure sensor can be maintained even after repetitive loading/unloading cycles and the sensor has high stability and durability without any degradation, which may be attributed to a PDMS molding structure. For comparison with previously reported literature, the comparison of the electrical performances of our sensors with existing sensors is listed in Table S2.

### 3.4. The Elastic Performance of Pressure Sensor in Stretching Test

**Figure 4:**
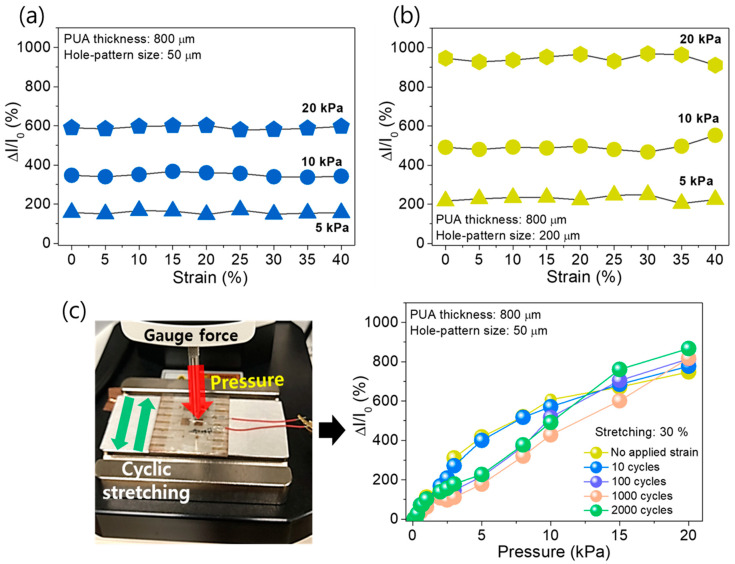
The strain durability response test of pressure sensors. The static responses of (**a**) D-50 and (**b**) D-200 pressure sensors in stretched state from 0% to 40%. The sensors pressed on 20, 10, and 5 kPa on customized stretching jig, respectively. (**c**) The picture of pressure measurement in gauge force (**left**) and a relative current change of pressure sensor after cyclic stretching test from without strain to the after 2000 cycles of 30% strain (**right**).

As indicated by the aforementioned experimental data, the various sensing properties such as sensitivity, sensing ranges, and linearity could be controlled by providing appropriate micro-patterned hole structures. The results may imply that a large-area stretchable pressure sensor system including site-specific sensing properties could be monolithically implemented by simply arranging different sized hole distribution, which can be more strategically applicable to specific targets. For example, some areas such as the fingertips more need high sensitivity rather than a wide sensing range, while it may be the opposite for other areas. This approach can enable the preparation of a variety of locally controlled sensing properties in a monolithic system in a scalable production manner. Additionally, to ensure the viability of the developed AgNW/PUA/AgNW sensor, the current variation under different strain conditions was measured. Figure 4a,b show the variation of ΔI/I_0_ when the sensors were strained in a range of 0~40% with the pressures of 5, 10, and 20 kPa. Here, the size of the holes was 50 μm (Figure 4a) and 200 μm (Figure 4b), and the thickness of PUA was fixed at 800 μm. As shown in both cases, the variation of ΔI/I_0_ was negligible or relatively small even at the 40% strained condition. This can be attributed to the good stretchability of each component, namely, the AgNW network, the PUA spacer, and the PDMS substrate. Here, it should be noted that, although the resistance of AgNW electrodes could be changed by the straining, the relative ratio of current changes in the devices can be negligible because, when the devices are strained, the initial current values (I_0_) are decreased and the current variation under pressure at the strained condition can be correspondingly decreased, possibly owing to the shrink of conductive pathways in the AgNW on the PUA sidewall by Poisson’s ratio. Therefore, the micro-hole patterned stretchable pressure sensor, for example, with D-50 hole, exhibits reliable detectability of pressure during the stretching conditions (from 0 to 40% strain, shown in Figure 4a) with average relative current change of 590.82%, 349.27%, and 157.32% (20, 10, and 5 kPa, respectively) and standard deviation of 8.34%, 10.33%, and 8.21% (20, 10, and 5 kPa, respectively). Moreover, the D-200 pressure sensor shows the average relative current change of 945.65%, 494.04%, and 228.97% (20, 10, and 5 kPa, respectively) and standard deviation of 20.08%, 24.17%, and 14.10% (20, 10, and 5 kPa, respectively) in the measured data set from 0 to 40% strain (shown in Figure 4b). Additionally, the bending test was implemented under the applied pressure of 10 kPa, resulting in a stable response to different bending angles without the deterioration of the sensor’s performances (Figure S10). Figure 4c shows the picture of pressure measurement environment in the gauge force and the stretching jig (left) and the mechanical durability test to prove resilient capacity (right). Even after the cyclic stretching test (from 10 to 2000 cycles of 30% strain), the sensors indicate relatively reliable sensitivity, which may be attributed to the stable junction networks of AgNW with stretching conditions (the number of junctions may be no longer increased or decreased, but the junction position just moved according to the stretching cycle).

Although, in this research, thin-film structured stretchable unit sensors are mainly developed and investigated, a pressure sensor array composing of the APA unit sensors with the effectively structural-tuning design capable of controlling the sensing properties can detect positional pressure stimuli and multifunctional sensing features for large-area stretchable pressure sensor systems [45].

## 4. Conclusions

We have demonstrated a controllable and stretchable piezoresistive pressure sensor based on silver-nanowires as stretchable electrode and PUA as the photo-patternable characteristic controlling the layer. The sensor was implemented with an APA sandwiched architecture using simple bottom-up spin-coating methods and photo patterning by UV. In the device structure, the pressure sensing range and sensitivity are determined by controlling the hole diameter and thickness of the PUA insulation layer, exhibiting a sensitivity of 38.86 kPa^−1^ in the pressure range of 0~20 kPa and up to 171.5 kPa^−1^ in the pressure range of 0~5 kPa in a D-10/200 APA sensor. Moreover, the minimum sensing threshold pressure is 100 Pa and the maximum is 20 kPa with a fast response time (100 ms). Additionally, the fabricated sensor can operate even in 40% stretched state without large deterioration. From these experiments, it is noted that the carefully controlled piezoresistive pressure sensing area and device architecture could provide a facile route to locally adjusted sensitivity with appropriate sensing ranges. Consequently, these features enable efficient systems by organizing cells with different sensitivity in a single system for the specific applications of artificial skins, wearable electronic device, and robotic system.

## Supplementary Materials

The following are available online at https://www.mdpi.com/1424-8220/20/22/6588/s1, Figure S1: The electron microscope image of micro-patterned holes in sensor; Table S1: The specific electrical properties about APA pressure sensors; Table S2: The comparable results of the pressure sensors based on AgNW; Figures S2 and S3: Electrical properties and linear fits of hole patterned pressure sensors; Figure S4: Electrical properties and linear fits with different thickness of pressure sensors; Figures S5 and S6: The performance tendency of APA sensor in different hole size and thickness of PUA; Figure S7: The dynamic performance of the APA pressure sensor at 1 Hz; Figure S8: Electrical properties with different step pressure; Figure S9: The transmittance of the APA pressure sensor; Figure S10: Electrical properties in bending test; Figure S11: A measurement setup for the stretchable pressure sensor.
