# Supplementary material for: Locally Controlled Sensing Properties of Stretchable Pressure Sensors Enabled by Micro-Patterned Piezoresistive Device Architecture
Source: Sensors (Basel). 2020 Nov 18;20(22):6588. doi: 10.3390/s20226588 (PMC7698782; doi:10.3390/s20226588)
Supplement: Supplementary file 1 [file sensors-20-06588-s001.pdf]

Supplementary Information

Locally Controlled Sensing Properties of Stretchable Pressure Sensors Enabled by Micro-Patterned Piezoresistive Device Architecture

Jun Ho Lee[1†], Jae Sang Heo[2†], Keon Woo Lee[1], Jae Cheol Shin[1], Jeong-Wan Jo[3], Yong-Hoon Kim[2, 4*] and Sung Kyu Park[1*]

[1] Department of Electrical and Electronics Engineering, Chung-Ang University, Seoul 06974, Korea
[2] School of Advanced Materials Science and Engineering, Sungkyunkwan University, Suwon 16419, Korea.
[3] Electrical Engineering Division, Department of Engineering, University of Cambridge, 9 JJ Thomson Avenue, Cambridge, CB3 0FA, United Kingdom.
[4] SKKU Advanced Institute of Nanotechnology (SAINT), Sungkyunkwan University, Suwon 16419, Korea.
* Correspondence: Prof. Sung Kyu Park (skpark@cau.ac.kr) and Prof. Yong-Hoon Kim (yhkim76@skku.edu)

The electron microscope image of micro-patterned holes in the APA pressure sensor.

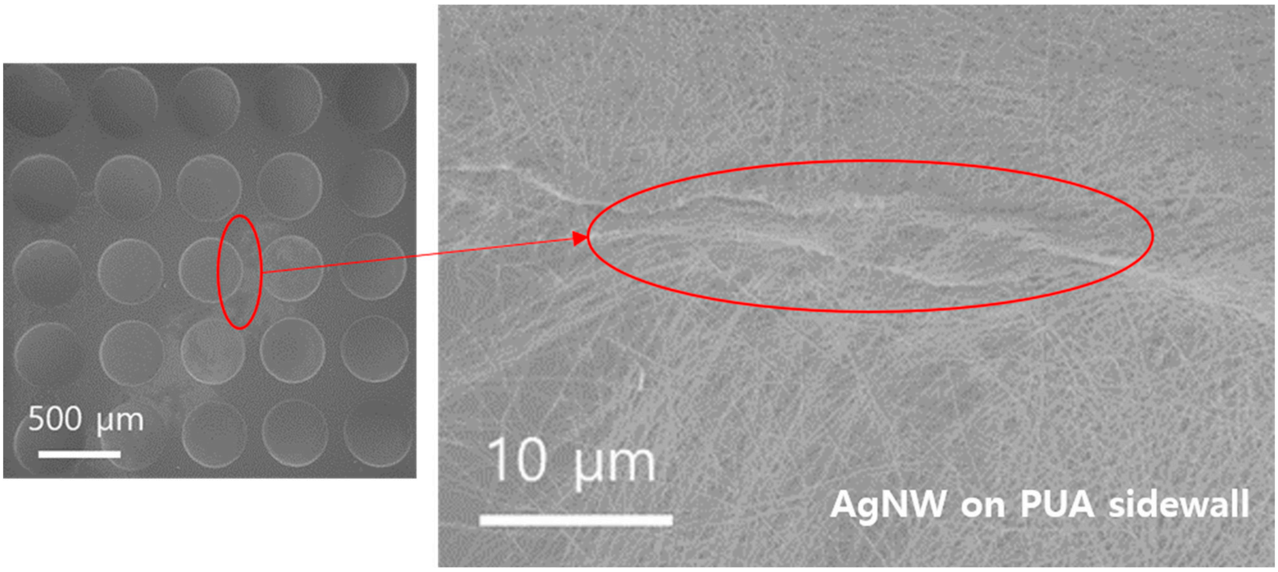

Figure S1. The SEM image of patterned hole and the specific AgNW image coated beside on patterned PUA wall.

Table S1. The specific electrical properties of the stacked APA pressure sensors.

Sensor	*Sensitivity (kPa^{-1})*	*Range (kPa)*	*Thickness (μm)*	*Density (hole area / μm^2)*
D-10	29.44	20	800	0.020274
D-50	60.7	10	800	0.018212
D-50	65.1	5	500	-
D-50	67.5	2.5	300	-
D-200	163.4	5	800	0.021806
D-10/50	38.86	20	800	0.035883
D-10/50	38.07	10	500	-
D-10/50	48.9	2.5	300	-
D-10/200	171.5	5	800	0.017663

Table S2. Comparison of the electrical properties of the existing AgNW-based pressure sensors.

Materials used	*Sensitivity (kPa^{-1})*	*Range (kPa)*	*Thickness (μm)*	*Resolution*	*Ref*
AgNF, AgNW, PEDOT:PSS	~ 1.78×10^{-3}	100	~ 1000	318 CPI	[1]
AgNW, Ecoflex, PDMS	0.57 ~ 1.62×10^{3}	~1300	~ 1000	X	[2]
AgNW, PU, PVP	5.54	60×10^{-3}	X	X	[3]
AgNW, PDMS	0.8 ~ 3.8	4.5	~ 1000	5x5 array	[4]
AgNW-PI, Ni, PVC	1.3294<br>0.6	0 ~ 0.6<br>0.6 ~ 80	X	X	[5]
AgNW, PUU, PDMS	X	250	~ 1000	3x3 array	[6]
AgNW, PDMS	20.08	18	Over 500	x	[7]

Electrical properties and linear fits of hole patterned APA pressure sensors

For more precise calculation of linearity of the pressure sensor, the linear fitting analysis was exhibited by Origin graph plot program. The linear equation has basic formula of y = a + b*x. As shown in figure S2, at first, D-10 sensor shows the best performance in linear characteristics that have the 31.24 of slope and 0.78 of standard error. Second, D-50 sensor shows the 40.35 of slope and 4.1 of standard error. At last, D-200 sensor shows 117.99 of slope and 17.32 of standard error. These results indicate the linearity to large pressure range (~20 kPa) is more correct in small size of diameter and the sensitivity is higher in large size of diameter pressure sensor.

With the same methods, the multi-mixed holes patterned pressure sensors linear fitting plots were shown in figure S3. D-10/50 sensor shows the 68.05 of slope and 3.9 of standard error. Also, D-10/200 sensor shows 177.57 of slope and 13.86 of standard error. These results indicate the D-10/200 sensor shows the highest-pressure sensing sensitivity with good linearity to low pressure range (~5 kPa) and D-10/50 sensor shows good linearity and sensitivity to middle pressure range (~8 kPa).

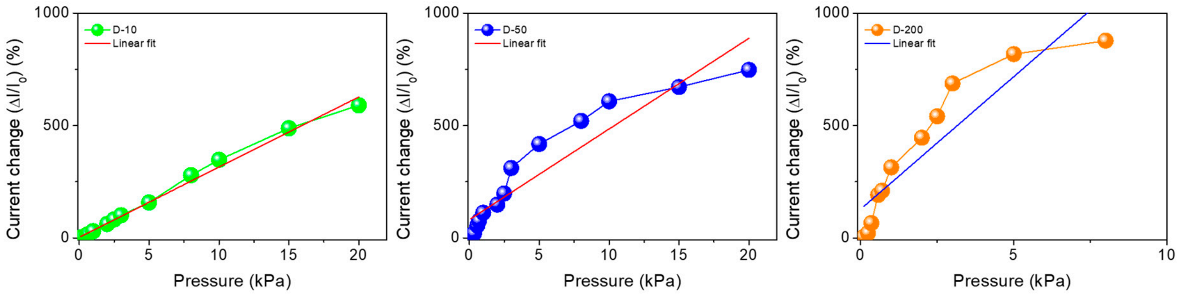

Figure S2. The linear fitting graph of single hole patterned pressure sensor of D-10, 50, 200.

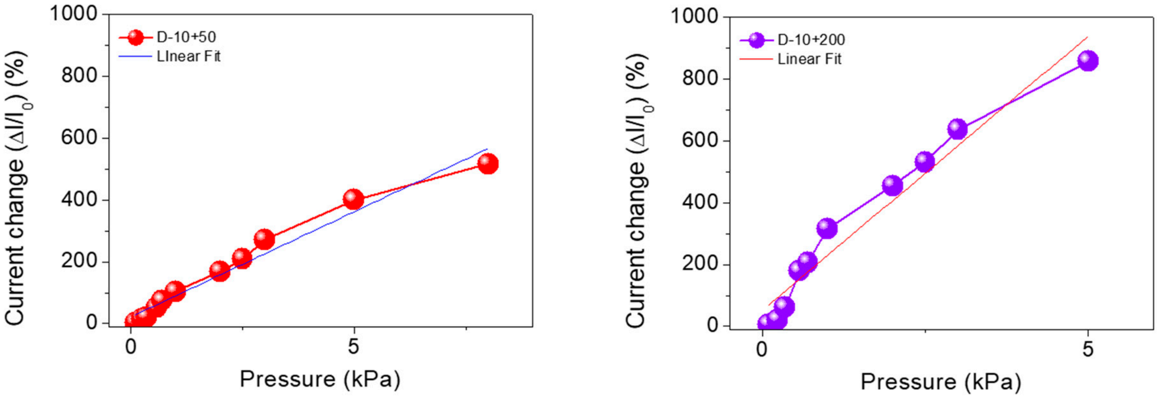

Figure S3. The linear fitting graph of multi-mixed holes APA pressure sensor of D-10/50, D-10/200.

Electrical properties and linear fits with different thickness of APA pressure sensors

Above mentioned methods, the D-50 pressure sensors linear fitting plots with different PUA thickness were shown in figure S4. T-300 sensor shows the 125.34 of slope and 10.7 of standard error. Also, T-500 sensor shows 40.57 of slope and 1.54 of standard error. At last, T-800 sensor shows 43.125 of slope and 1.67 of standard error. These results indicate the T-300 sensor shows the highest-pressure sensing sensitivity with good linearity to low pressure range (~1 kPa) and T-500 sensor shows good linearity and sensitivity to middle pressure range (~5 kPa).

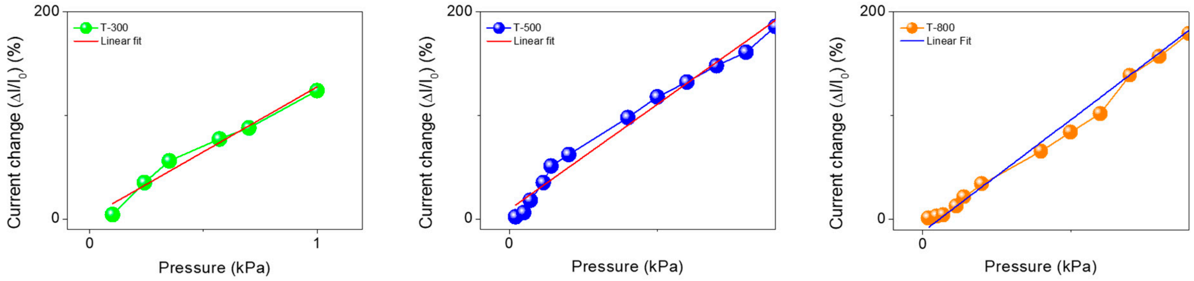

Figure S4. The linear fitting graph of D-50 APA pressure sensor with different PUA thickness.

The performance tendency of APA sensor in different hole size and thickness of PUA

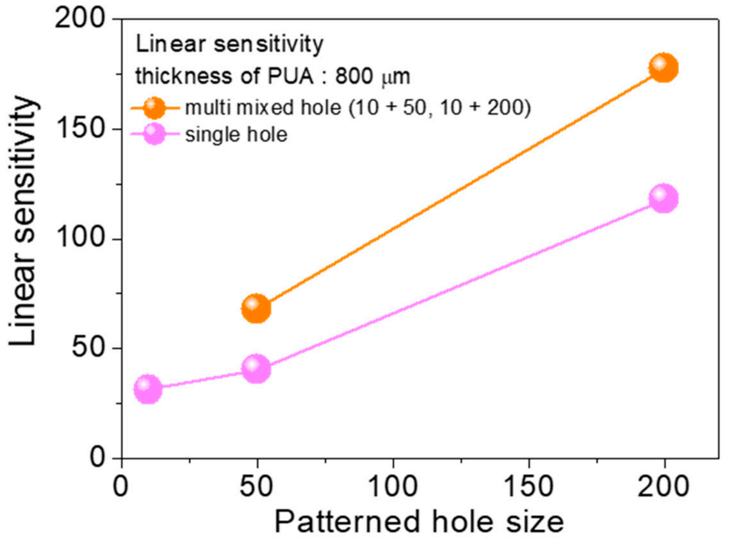

Figure S5. The tendency of linear sensitivity in different hole sizes and patterns (single or multi-mixed).

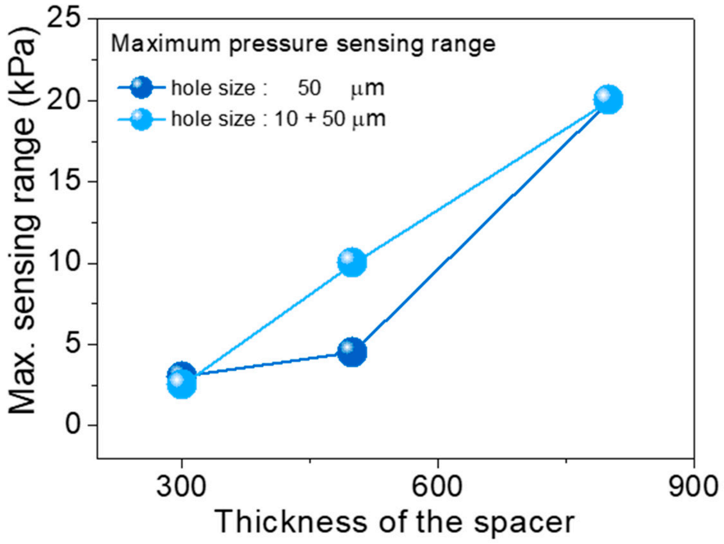

Figure S6. The tendency of maximum pressure sensing range in different thickness of spacer.

The dynamic performance of the APA pressure sensor at 1Hz

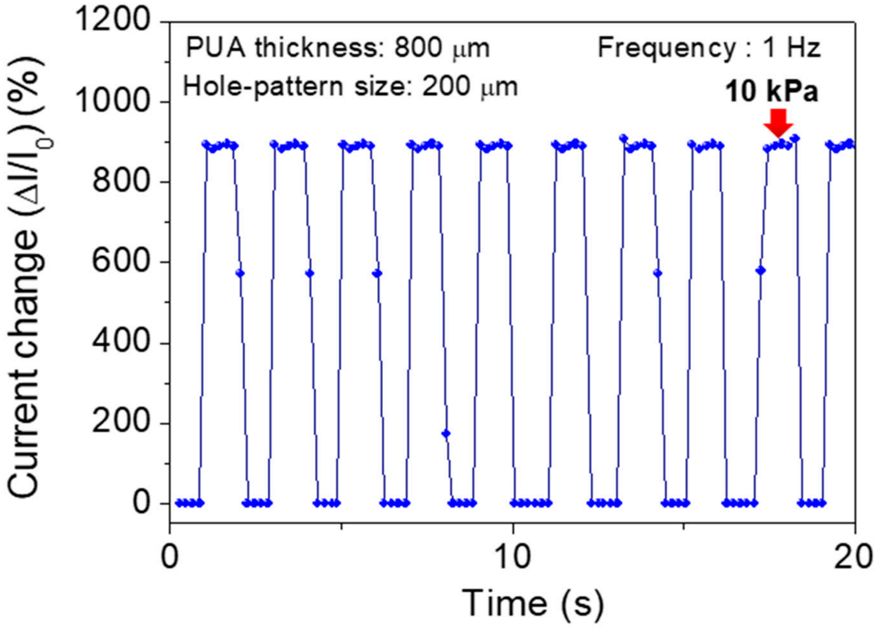

Figure S7. A dynamic response to the repetitive applied pressure of 10 kPa for 20 s at the frequency of 1 Hz.

Electrical properties with different step by step pressure sensing of the APA pressure sensors

For more precise dynamic performance, the step up & down test was implemented to D-10, T-800 APA pressure sensor. The pressure was applied from 0, 2.5, 5,10,15 and 20 kPa until about 2 seconds in respect step, then applied from 20 kPa to 0 kPa as the same way with step-up level. As shown in figure S8, the result shows almost symmetric characteristics

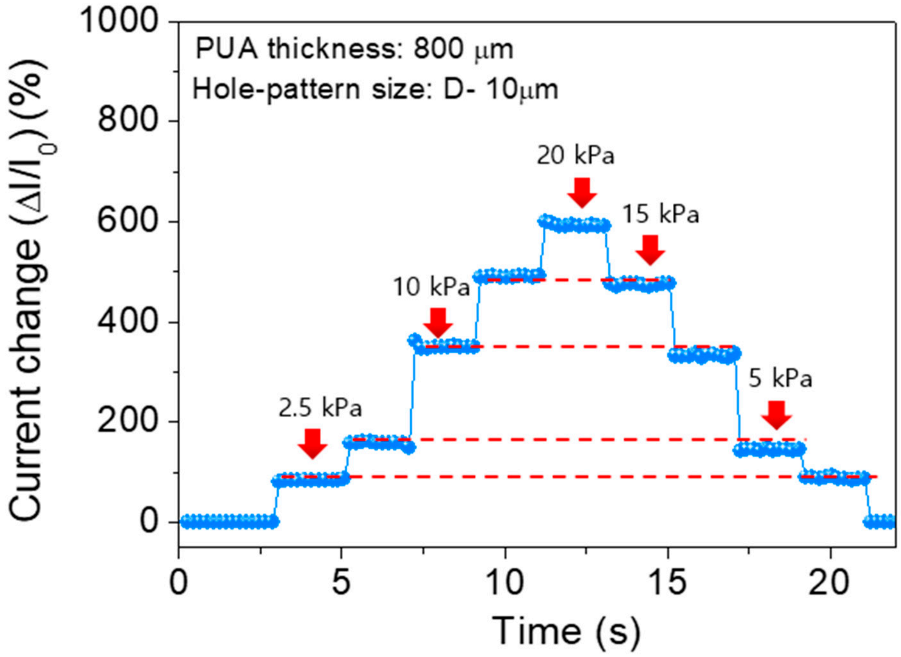

Figure S8. Change in current of the APA sensor fabricated with D-10 and T-800 under loading pressure of 2.5 kPa, 5.0 kPa, 10 kPa, 15 kPa, and 20 kPa.

Transmittance of the APA pressure sensors

The measured transmittance indicates APA sensor/PDMS, APA sensor/glass respectively. The average transparency shows the 56.89 and 45.94 %, respectively. Actually, the sensor shows not high transparency because the multiple coating of AgNW layer (twice as top, twice as bottom electrode) on PDMS. However, the area without AgNW layer, shows good transparency about 84.98%.

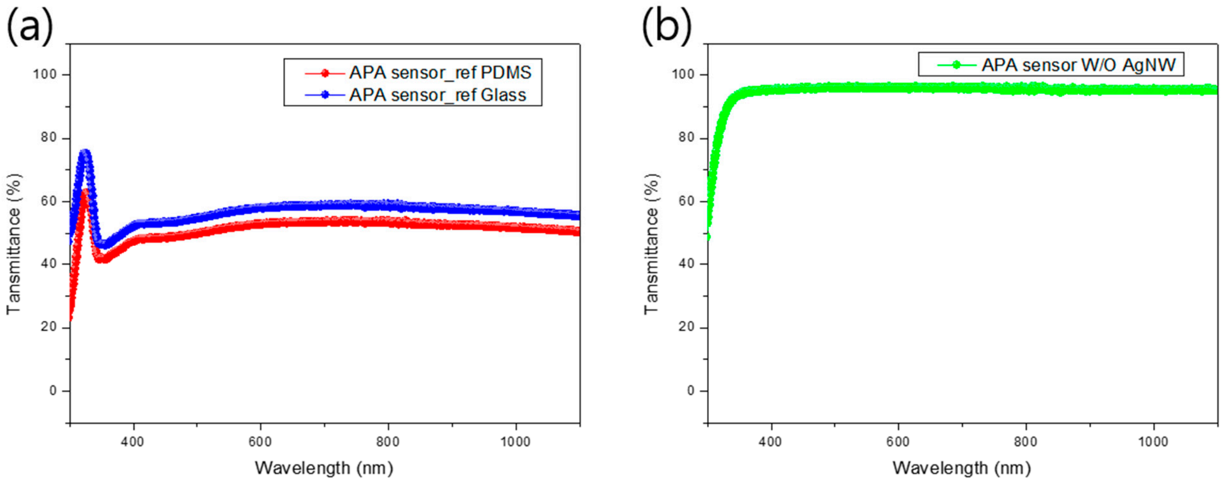

Figure S9. The transparency of (a) the APA pressure sensor and (b) the PDMS substrate.

Electrical properties of APA pressure sensors in bending test

The APA pressure sensor was bent on thin spatula (~ Φ 2.5, 5mm) (figure S10 left) and with specific angle (30, 60 and 90°). As shown in figure S10 (right), the APA sensor operates like normal state even in bent without deterioration.

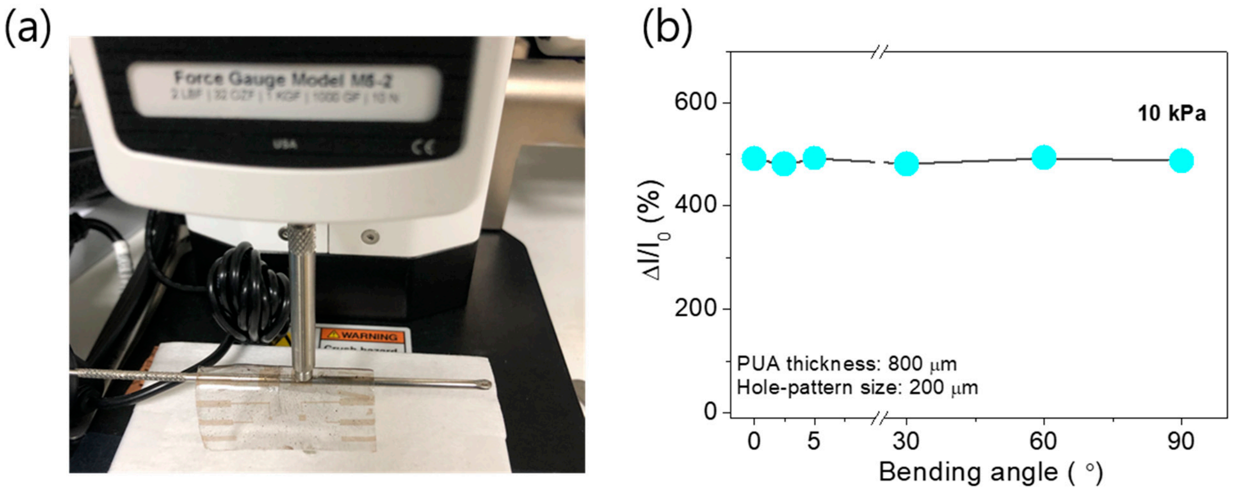

Figure S10. (a) The bending test and (b) the corresponding relative change in current of the APA sensor devices during different bending with the applied pressure of 10 kPa.

A measurement setup for the stretchable pressure sensor.

The measurement equipment of sensor array consists of four main components such as Keithley 2636B (as a dual channel sourcemeter), and a homemade stretching endurance jig tester and a customized gauge force system. Typically, Keithley 2636B are measures an electrical resistance of each sensor. After measuring an electrical resistance, using the data acquisition software (DAQ; snM co,. Ltd) installed in PC, initial current and relative change in current for the pressure sensor were automatically saved and displayed. Figure S11 shows the photograph of the pressure sensor connected with measuring system in customized pressure gauge force.

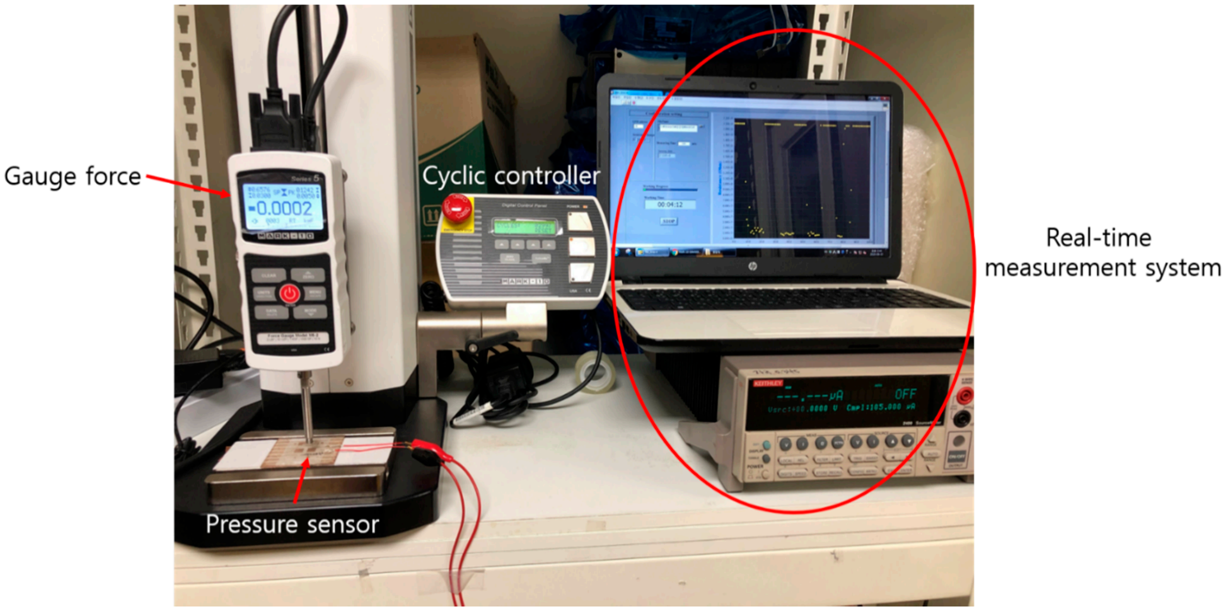

Figure S11. A measurement equipment and DAQ system for the pressure sensor.

References

1. An, B. W.; Heo, S.; Ji, S.; Bien, F. & Park, J. Transparent and flexible fingerprint sensor array with multiplexed detection of tactile pressure and skin temperature. *Nat. Commun.* **2018** 9, *2458*.
2. Yao, S. & Zhu, Y. Wearable multifunctional sensors using printed stretchable conductors made of silver nanowires. *Nanoscale* **2014**, *6*, 2345–2352.
3. Wang, J.; Jiu, J.T.; Nogi, M.; Sugahara, T.; Nagao, S. J.; Koga, H.; He, P.; Suganuma, K. A highly sensitive and flexible pressure sensor with electrodes and elastomeric interlayer containing silver nanowires. *Nanoscale* **2015**, *7*, 2926–2932.
4. Joo, Y.; Byun, J.; Seong, N.; Ha, J; Kim, H.; Kim, S.; Kim, T.; Im, H.; Kim, D.; Hong, Y. Silver nanowire-embedded PDMS with a multiscale structure for a highly sensitive and robust flexible pressure sensor. *Nanoscale* **2015**, *7*, 6208–6215.
5. Li, H., Ding, G. & Yang, Z. A high sensitive flexible pressure sensor designed by silver nanowires embedded in polyimide (AgNW-PI). *Micromachines* **2019**, *10*.
6. You, B.; Han, C. J.; Kim, Y., Ju; B. K. & Kim, J. W. A wearable piezocapacitive pressure sensor with a single layer of silver nanowire-based elastomeric composite electrodes. *J. Mater. Chem. A,* **2016**, 10435–10443.
7. Mao, Y.; Ji, B.; Chen, G.; Hao, C.X.; Zhou, B. P.; Tian, Y.Q. Robust and Wearable Pressure Sensor Assembled from AgNW-Coated PDMS Micropillar Sheets with High Sensitivity and Wide Detection Range. *ACS Appl. Nano Mater* **2019**. *2*, 3196–3205.
